# Overexpression of IGF2 and IGF2 receptor in malignant solitary fibrous tumor with hypoglycemia: a case report

**DOI:** 10.1186/s40792-018-0508-2

**Published:** 2018-08-30

**Authors:** Yusuke Arakawa, Hidenori Miyake, Hidehisa Horiguchi, Taku Inokuchi, Naoki Hino, Takashi Ogasawara, Takeshi Kuroda, Shinichi Yamasaki

**Affiliations:** 1 Department of Surgery, Tokushima Municipal Hospital, Kitajyosanjima 2-34, Tokushima, 770-0812 Japan; 2 Department of Pathology, Tokushima Municipal Hospital, Kitajyosanjima 2-34, Tokushima, 770-0812 Japan; 3 Department of Endocrinology, Tokushima Municipal Hospital, Kitajyosanjima 2-34, Tokushima, 770-0812 Japan

**Keywords:** No islet cell tumor hypoglycemia, Solitary fibrous tumor, Insulin-like growth factor-2, Insulin-like growth factor-2 receptor

## Abstract

**Background:**

Solitary fibrous tumor (SFT) is a prototypical mesenchymal neoplasm that induces non-islet cell tumor hypoglycemia (NICTH) due to overproduction of insulin-like growth factor 2 (IGF2). We here report the case of a malignant SFT associated with a hypoglycemia attack.

**Case presentation:**

An 81-year-old man with a large subphrenic mass presented with hypoglycemia and loss of consciousness. His serum insulin and IGF1 levels were relatively low, suggesting an excessively high serum IGF2 levels. Preoperative Western blotting of serum confirmed the overproduction of high-molecular-weight IGF2. After total tumor resection, the patient recovered from hypoglycemia without the need for further treatment. Histological examination revealed proliferation of spindle cells and frequent nuclear mitoses with STAT6 and CD34 immunoreactivity, which led to the diagnosis of malignant SFT. IGF2 was strongly upregulated in the tumor upon immunohistochemistry, consistent with the report of NICTH. In addition, the tumor expressed IGF2 receptor (IGF2R) but not IGF1R.

**Conclusions:**

The present results indicate that the tumor co-expressed IGF2 and IGF2R. IGF2R has not previously been recognized as a tyrosine kinase receptor participating in cell signal transduction. Thus, further case series are required to determine whether IGF2R overexpression reflects the action of an unknown autocrine/paracrine system involving IGF2 for cell proliferation or for the scavenging and degradation of IGF2.

## Background

Solitary fibrous tumor (SFT) is a rare mesenchymal tumor preferentially occurring in deep soft tissue. Most SFTs are benign, and total tumor resection yields a favorable outcome for patients. However, several of these tumors are highly aggressive and are frequently recurrent and invasive. Although histological examination is a relatively reliable prognostic method, it cannot always effectively predict the behavior of SFTs; furthermore, no definite markers of tumor progression have been identified to date. CD34 has been used as a conventional marker of SFT; however, some mesenchymal tumors histologically mimicking SFT also express CD34. Moreover, obliteration or downregulation of CD34 has been reported in some SFTs [1, 2]. Therefore, it is difficult to distinguish some malignant SFTs from other sarcomas. Recently, the *NAB-STAT6* fusion gene was recognized as a key factor contributing to SFT tumorigenesis [3]. In addition, STST6 immunoreactivity was established as a more sensitive and specific diagnostic biomarker than CD34 for SFT [1].

The peptides insulin-like growth factors 1 and 2 (IGF1/2) are structurally homologous to insulin, and they participate in serum glucose metabolism. IGF2 overexpression in neoplasms causes non-islet cell tumor hypoglycemia (NICTH). SFT is also known to be frequently associated with NICTH, owing to excessive IGF2 production [4, 5]. The paraneoplastic syndrome of hypoglycemia due to IGF2 hyper-secretion by pleural SFT is more commonly known as “Doege-Potter syndrome” [6, 7]. As a receptor ligand, IGF2 binds to IGF1 receptor (IGF1R), IGF2 receptor (IGF2R), and insulin receptor (IR) [8]. Although IGF2/IGF1R and IGF2/IR complexes primarily activate intracellular signal transduction for cell proliferation [9], some studies have reported that IGF2/IGF2R complex plays a role in cancer progression [10]. Moreover, the association of IGF2R/IGF2 with cancer progression appears controversial.

Herein, we report a case of malignant SFT associated with a hypoglycemia attack, and we discuss the associations among IGF2, IGF1R, and IGF2R in SFT based on analyses of the tumor in this case.

## Case presentation

**Fig. 1:**
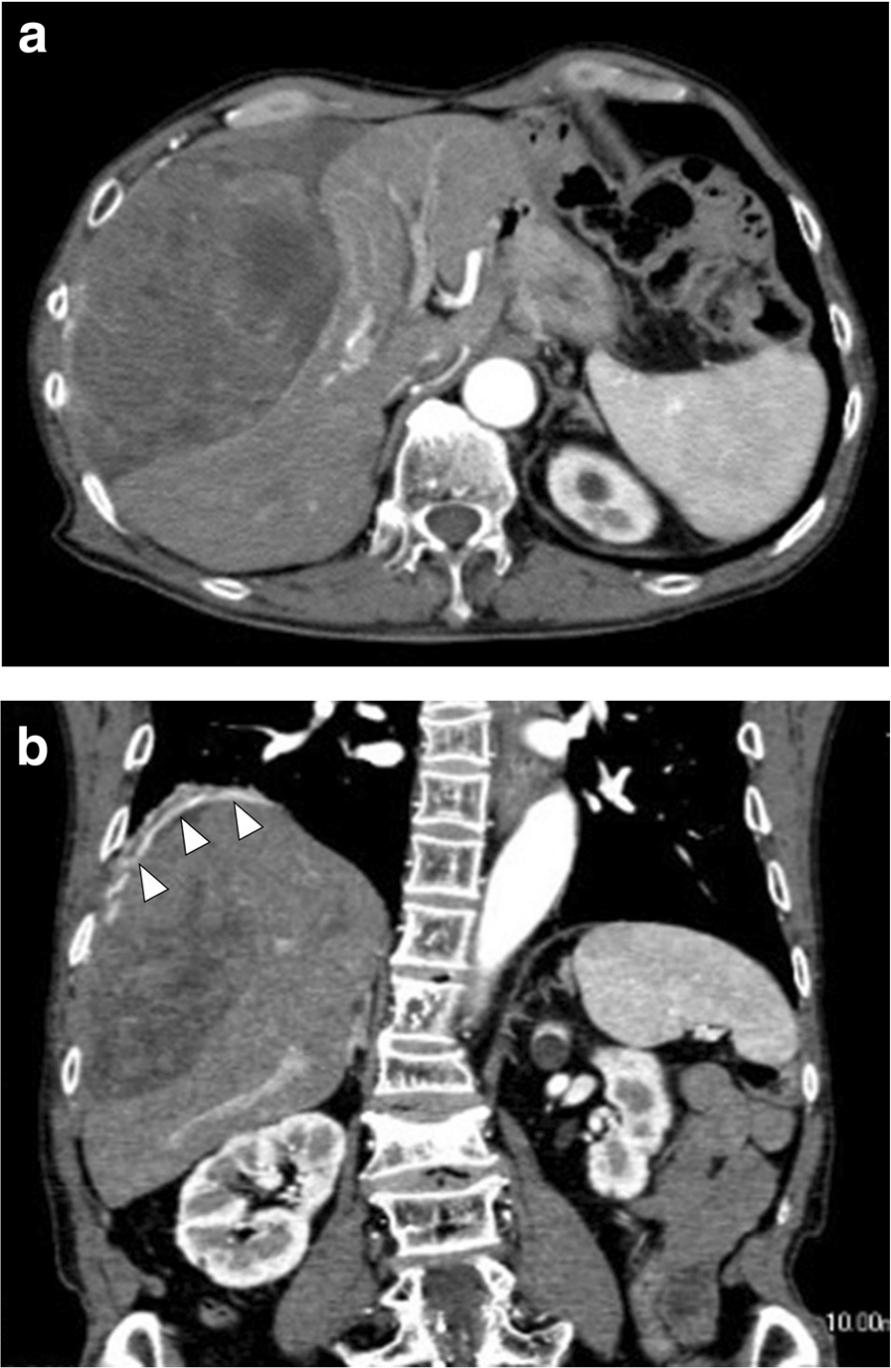
Computed tomography image showing a large subphrenic tumor. The heterogeneously enhanced tumor was well demarcated from the liver (**a**). The feeding artery originated from the diaphragm (arrowhead) (**b**)

**Fig. 2:**
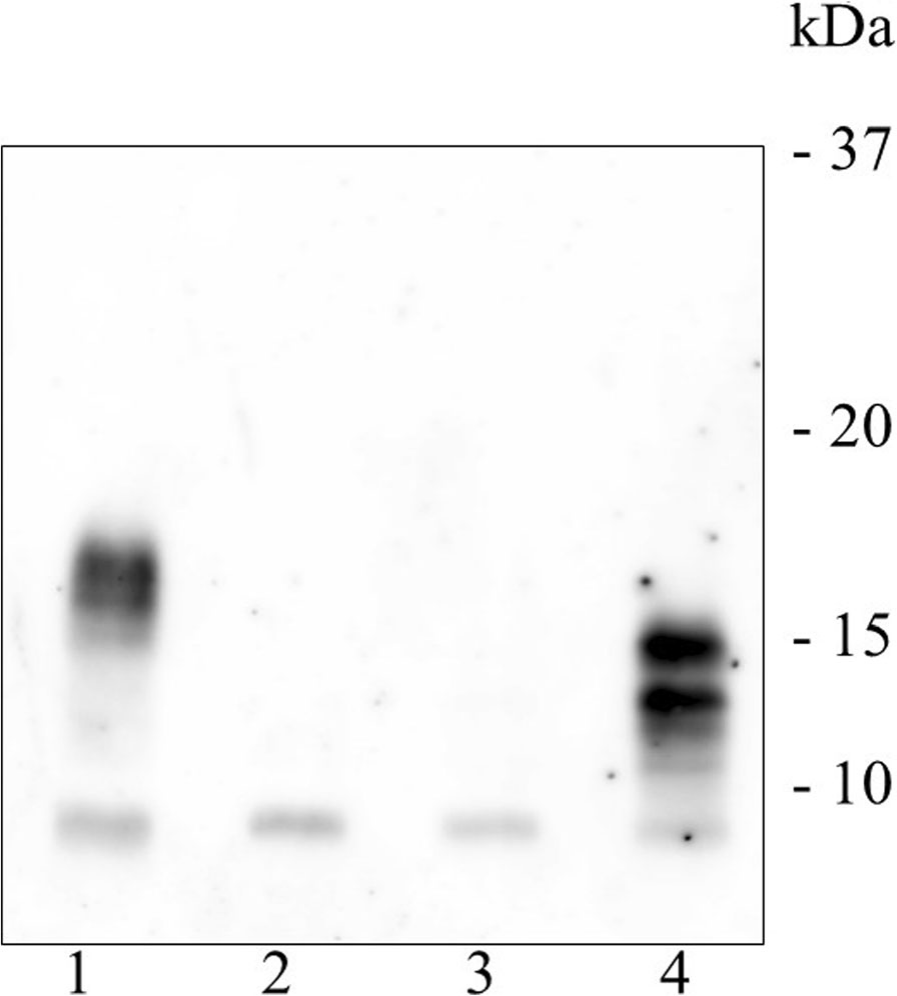
Western blot analysis of serum. Compared to healthy control IGF2 (lane 3) and a previous IGF2-producing tumor (lane 4), preoperative serum (lane 1) of the patient contained high-molecular-weight IGF2, designated “big IGF2.” Postoperative serum (lane 2) of the patient showed IGF2 recovery within the physiological range

**Fig. 3:**
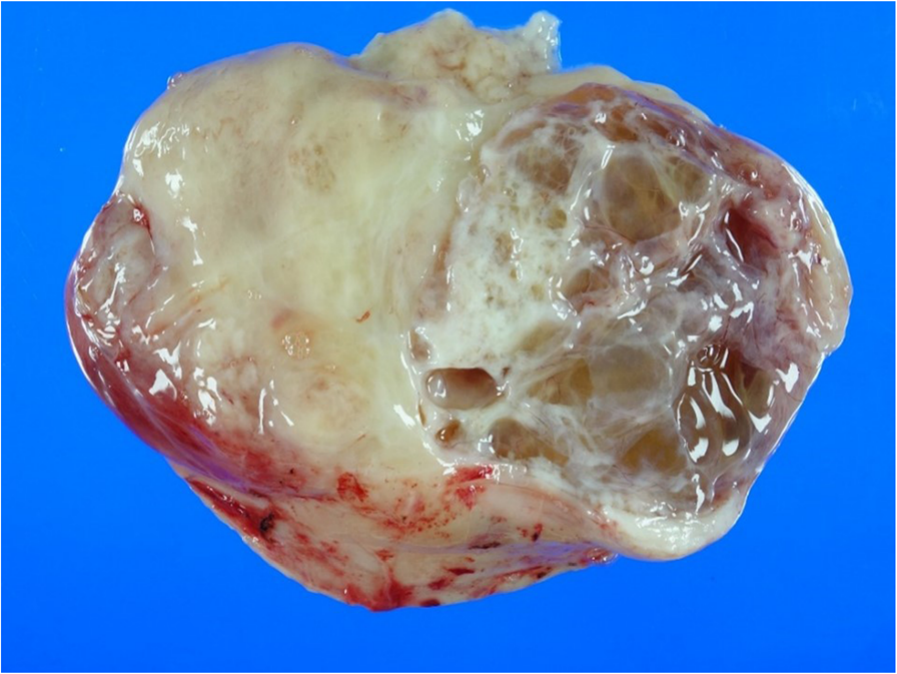
Macroscopic analysis of the malignant solitary fibrous tumor. The tumor displayed a firm, ivory-like appearance and partly cystic and myxoid resected surface

**Fig. 4:**
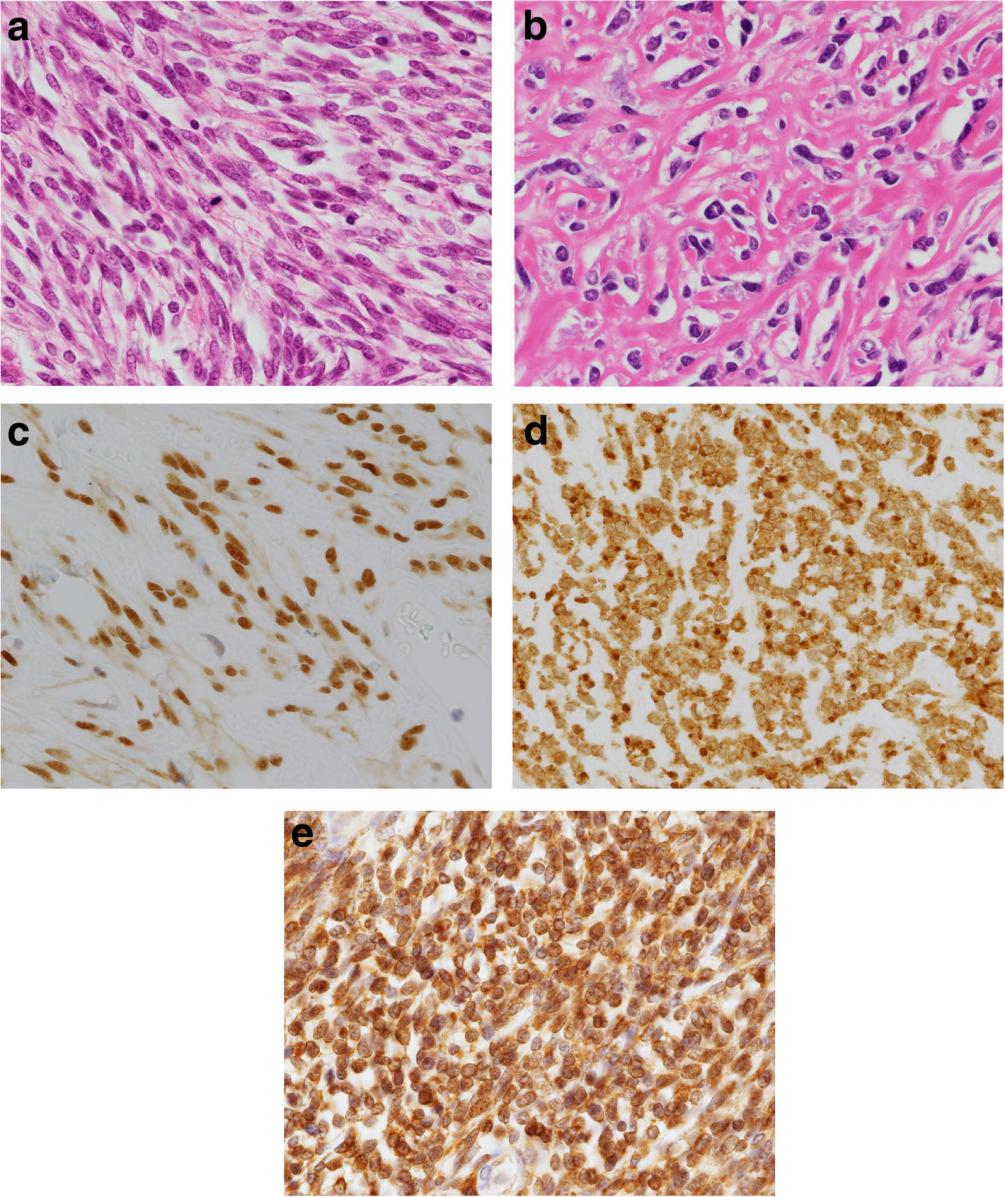
Histology and immunohistochemistry of the malignant solitary fibrous tumor (SFT). Atypical spindle cells proliferated in the major hypercellular area (**a**). The tumor showed typical SFT-like features with ovoid nuclei and a thick collagenous stroma (**b**). Tumor cells demonstrated immunoreactivity for STAT6, a hallmark of SFT (**c**). The tumor expressed IGF2. Characteristic dot-like reactivity corresponding to the Golgi complex was apparent (**d**). The tumor also showed immunoreactivity for IGF2R (**e**)

An 81-year-old man presented with abdominal discomfort. Computed tomography imaging revealed a large tumor with intermediate signal intensity, showing heterogeneous contrast enhancement in the subphrenic area (Fig. 1a) and the feeding artery originated from the diaphragm (Fig. 1b). Within several days, he suddenly experienced loss of consciousness. Serum examination indicated hypoglycemia (glucose levels, 18 mg/dL). Insulin (1.05 μIU/mL), C-peptide (0.71 ng/ml), and IGF1 (39 ng/mL) levels were all relatively low but still within the physiological range. Western blot analysis of the patient’s serum revealed overexpression of high-molecular-weight IGF-2 designated “big IGF-2” (Fig. 2). Along with glucose compensation, the patient underwent surgery for total tumor resection. In the operative view, the large tumor appeared to compress the right lobe of the liver without invasion. The feeding artery originating from the diaphragm was ligated and divided. A part of the diaphragm was resected with autosuture owing to firm adherence of the diaphragm to the artery. The tumor was then dissected along the liver surface without simultaneous resection of any other organs, and a tumor-free margin was achieved macroscopically. The tumor measured 34 cm at the major axis and weighed 1350 g and was well demarcated by a fibrous membrane. The resected surface was elastic, firm, had an ivory-like appearance, and was multilobulated with trabeculation. The solid component was predominant, and a small myxoid component was also noted (Fig. 3). Microscopically, spindle cells generally constituted the tumor without specific cellular arrangement in the solid component (Fig. 4a). A few areas in this component demonstrated keloid-like collagenous stroma (Fig. 4b) and stag horn-like vessels. However, some areas with myxoid appearance upon gross examination revealed a multi-cystic formation by cavernous hemangioma-like septa. Karyokinesis was detected in more than 0/10 high-power fields in the hypercellular area. Necrosis was obscure. Immunohistochemically, the tumor cells expressed STAT6 (Fig. 4c) and CD34. The highest Ki-67 labeling index was 15%. These results confirmed that the tumor was a malignant SFT. In addition, the tumor cells exhibited cytoplasmic IGF2 expression (Fig. 4d), specifically with paranuclear dot-like reactivity. The tumor cells were positive for IGF2R (Fig. 4e) but negative for IGF1R. Since surgery, the patient has been free from tumor recurrence and hypoglycemia for more than 24 months.

## Conclusions

SFT displays various histological features such as fat-forming, myxoid, and even epithelioid characteristics. Therefore, immunohistochemistry is necessary for pathological diagnosis. The present tumor showed an undifferentiated sarcoma-like histology in the numerous areas examined and a typical SFT histology in smaller components. Nevertheless, the tumor demonstrated strong immunoreactivity for STAT6, thereby indicating SFT, whereas CD34 was only weakly expressed.

Previously reported cases of SFTs with NICTH have demonstrated an upregulation of a precursor form of IGF2, or “big IGF2” [11, 12]. However, “big IGF2” levels can only be quantified in limited laboratories. An alternative method of detection of the IGF2 overproduction is appropriate suppression of insulin and C-peptide but inappropriately low GH and IGF1 levels in blood [8, 13]. Thus, although the measurement of serum IGF2 is still challenging, it has now become easier to detect IGF2-producing SFTs because numerous SFTs conveniently disclose IGF2 production upon immunohistochemistry. IGF2 was overexpressed in the present tumor upon both Western blot analysis and immunohistochemistry.

The relationship between IGF2 expression and tumor grade is unclear. Some reports have suggested an association between IGF2 expression and metastasis [14] or poor prognosis [8]. However, SFTs frequently express IGF2 and numerous cases involve an indolent clinical course. Thus, besides protein assessment, quantitative analysis targeting mRNA is required for precise prediction of the prognosis and to determine the degree of IGF2 production in the future.

IGF2R has been recognized as a multifunctional receptor but not as a tyrosine kinase receptor [15, 16]. However, an in vitro study [17] revealed that IGF2R knockdown significantly reduced IGF2-related ERK1/2 phosphorylation, suggesting that the complex of IGF2 and IGF2R participates in cancer cell proliferation. In the present case, the tumor expressed both IGF2 and IGF2R; however, IGFR1 was unexpectedly downregulated. However, it remains unknown whether IGF2/IGF2R exert their effects through an autocrine/paracrine mechanism or whether IGF2R simply plays a role as a scavenger of IGF2 in SFTs. Further studies involving a larger patient cohort are necessary to clarify this mechanism.

In conclusion, the present tumor determined to be malignant SFT demonstrated high IGF2 production corresponding to NICTH. Although IGF1R was not expressed in the tumor, IGF2R was overexpressed. These results suggest that the interaction between IGF2 and IGF1R is not involved in tumor progression in SFT. Therefore, the role of the IGF2/IGF2R complex in cancer progression should be investigated in further studies.

## Abbreviations

IGF1/2: Insulin-like growth factors 1 and 2
IGF1R/2R: Insulin-like growth factor receptors 1 and 2
IR: Insulin receptor
NICTH: Non-islet cell tumor hypoglycemia
SFT: Solitary fibrous tumor
